# Validating the CogSleep Screener in older adults at a memory and cognition clinic

**DOI:** 10.1111/jsr.14355

**Published:** 2024-09-30

**Authors:** Shawn Dexiao Kong, Zoe Menczel Schrire, Ping Hsiu Lin, Simone Simonetti, Nathan Cross, Loren Mowszowski, Catriona Ireland, Ivana Rosenzweig, Sharon L. Naismith

**Affiliations:** ^1^ Healthy Brain Ageing Program, Brain and Mind Centre University of Sydney Camperdown New South Wales Australia; ^2^ Charles Perkins Centre University of Sydney Camperdown New South Wales Australia; ^3^ School of Psychology, Faculty of Science University of Sydney Camperdown New South Wales Australia; ^4^ Plasticity Centre, Department of Neuroimaging, Institute of Psychiatry, Psychology and Neuroscience (IoPPN) King's College London London UK

**Keywords:** memory clinic, mild cognitive impairment, older adults, psychometrics, sleep disturbances

## Abstract

While sleep disturbances are prevalent in older people and are linked with poor health and cognitive outcomes, screening for the range of sleep disturbances is inefficient and therefore not ideal nor routine in memory and cognition clinic settings. We aimed to develop and validate a new brief self‐report questionnaire for easy use within memory and cognition clinics. The design for this study was cross‐sectional. Older adults (aged ≥50 in Sydney, Australia) were recruited from a memory and cognition research clinic. Participants (*N* = 497, mean age 67.7 years, range 50–86, 65.0% female) completed a comprehensive medical, neuropsychological, and mental health assessment, alongside self‐report instruments, including existing sleep questionnaires and a new 10‐item sleep questionnaire, the CogSleep Screener. We examined the factor structure, convergent validity, internal consistency, and discriminant validity of this novel questionnaire. Using exploratory factor analysis, a 3‐factor solution was generated highlighting the factors of *Insomnia*, *Rapid Eye Movement* (*REM*) *Symptoms* and *Daytime Sleepiness*. Each factor was significantly correlated with currently used sleep questionnaires for each subdomain (all Spearman rho >0.3, all *p* < 0.001), suggesting good convergent validity. Internal consistency was also good (Revelle's ω = .74). Receiver operating characteristic curves showed good discriminative ability between participants with and without sleep disturbances (all area under curve >0.7, all *p* < 0.01). The CogSleep Screener has good psychometric properties in older to elderly adults attending a memory and cognition clinic. The instrument has the potential to be used in memory clinics and other clinical settings to provide quick and accurate screening of sleep disturbances. [Correction added on April 2025, after first publication: The number of participants has been updated and associated statistics have been updated]

## 1 INTRODUCTION

Sleep disturbances encompass conditions that disrupt a person's ability to fall or remain asleep, or pose difficulty sleeping due to exogenous influences such as poor sleep hygiene (Alfano et al., 2007). Symptoms can include frequent waking during the night or early morning waking, which can result in a shorter or longer than optimal sleep duration or feeling unrefreshed after sleep. Chronic sleep disturbances can lead to daytime fatigue, decreased cognitive abilities, and other negative health outcomes (Halperin, 2014). Importantly, sleep disturbances are linked with poor quality of life (Reimer & Flemons, 2003) and other negative health outcomes such as depression onset and recurrence in older adults (Cho et al., 2008).

As individuals advance in age, they are likely to encounter changes to the quantity and quality of their sleep, which may be particularly noticeable in those who suffer from neurodegenerative disorders (Anderson & Bradley, 2013). This issue is of clinical importance, given the projected increases in the global proportion of older adults (>60 years), which were expected to almost double from 12% to 22% between 2015 and 2022 (World Health Organization, 2015). There is also mounting evidence that sleep disturbances and dementia have a bi‐directional relationship; presence of disordered sleep increases the risk for dementia, as well as the risk for sleep disorders after the development of Alzheimer's Disease (Irwin & Vitiello, 2019). Furthermore, poor sleep is linked to a range of short term and long term negative outcomes such as reduced alertness (de Mello et al., 2013), weakened overnight memory consolidation (Cellini, 2017), and impaired daily functioning (Szentkirályi et al., 2009).

The terminology "sleep disturbance" is fairly heterogeneous. In terms of sleep disorders, insomnia (difficulty falling asleep or maintaining sleep) and obstructive sleep apnea (a disorder of altered breathing during sleep) are common (i.e., ranging from 50%–70%) in older adults (Patel et al., 2018) and are linked to dementia longitudinally (Blackman et al., 2021). While less common, idiopathic rapid eye movement behaviour disorder (RBD) can be a strong predictor of developing dementia with Lewy bodies or Parkinson's disease. Studies have shown that 80% of individuals diagnosed with idiopathic RBD will likely develop a neurodegenerative disease, such as dementia with Lewy bodies or Parkinson's disease, within 10 years of diagnosis (Bramich et al., 2022). With regards to more general sleep disturbance, short sleep duration predicts longitudinally the development of dementia (Sabia et al., 2021) and both sleep duration and sleep latency are associated with the presence of brain beta‐amyloid (Ab) (Brown et al., 2016; Winer et al., 2021) which may vary according to age (Naismith et al., 2022). In those with mild cognitive impairment (MCI), poor sleep quality (McKinnon et al., 2014), nocturnal awakenings (Naismith et al., 2010), changes to sleep macro‐architecture (D'Rozario et al., 2020), and circadian timing (Naismith et al., 2014) are especially pronounced. Given that the range of sleep disturbances and disorders in older adults may have differential underpinnings and treatment options, there is a need for a tool to efficiently detect the diverse presentations of sleep disturbances in the clinical setting.

In addition to the range of sleep disturbances, assessment methods vary. While polysomnography (PSG) is currently considered the gold standard for sleep assessment, it has been criticised for lacking ecological validity (Sánchez‐Ortuño et al., 2010) and having first night effects (Newell et al., 2012), and is inaccessible to many (Boulos et al., 2019) due to long wait times, costs, and the need to access specialist laboratories. Actigraphic assessment of sleep–wake cycles offers greater ecological validity and is more accessible, but still requires equipment, monitoring, concurrent sleep diary reporting as well as subsequent scoring and interpretation. It can therefore be impractical in clinical environments that lack such equipment or expertise (Chow et al., 2016).

Indeed, in clinical practice, self‐report methods are quick and easy to administer and score (Buysse et al., 2010). Currently, however, clinicians must utilise and collate sleep information from several disparate measures, which presents time and feasibility barriers for routine use. In addition, data from the various measures capture different timeframes (e.g., from a week to a month), or are unsuitable for the memory clinic setting as they have been developed and validated for use in healthy people, or for use in sleep clinics (e.g., insomnia, OSA). Importantly, there is a need to develop an instrument to “flag” the most alarming sleep disturbances symptoms that requires further investigation which may be time consuming or may incur additional costs (e.g., see a sleep physician or get referred to a polysomnography study).

Therefore, the current study aims to validate a novel questionnaire ‐ The CogSleep Screener to screen for sleep symptoms for use in people presenting to clinical settings with early cognitive decline. The questionnaire was designed to provide quick, accurate, and multifactorial screening of the various forms of sleep disturbances in older adults experiencing early cognitive decline, including those with subjective cognitive decline (SCD), MCI, and early dementia. [Correction added on April 2025, after first publication: The aim and rationale of the study have been further clarified.]

## 2 METHODS

### 2.1 PARTICIPANTS

Individuals were recruited from the Healthy Brain Ageing (HBA) Clinic, a specialist memory and cognition clinic in Sydney, Australia. The clinic accepts general practitioner or specialist referrals for individuals aged ≥50 years with concerns about new‐onset cognitive and/or mood changes and a Mini‐Mental State Examination (MMSE) score of ≥20. Participants were excluded from the clinic if they had: (i) insufficient English language proficiency to complete neuropsychological assessments; (ii) pre‐existing diagnosed intellectual disability; (iii) history of stroke, head injury with loss of consciousness persisting for >30 min, or other neurological disorders (e.g., epilepsy); (iv) history of non‐affective psychiatric disorder (e.g., schizophrenia); or (v) current substance dependence or abuse. For this study, consecutive participants attending the clinic between 2017 and 2024 were invited to complete the CogSleep Screener questionnaire. Written informed consent was obtained for all participants and this research was approved by the Human Research Ethics Committee of The University of Sydney.

### 2.2 Assessments

#### 2.2.1 Clinician assessments

Medical: Using a semi‐structured interview, a geriatrician recorded demographic information and completed a full medical, sleep, mental health, and medication history. For descriptive purposes, the geriatrician also administered the MMSE and the Cumulative Illness Rating Scale Geriatric Version (CIRS‐G; Miller et al., 1992) to measure global cognition and medical burden respectively.

Mental health and sleep: The mental health assessment was conducted by a trained psychologist who used the Mini‐International Neuropsychiatric Interview (MINI) (Sheehan et al., 1998) to ascertain the presence or absence of current and lifetime major depression. A semi‐structured interview was used to probe for various sleep concerns and disorders.

Neuropsychological: A clinical neuropsychologist administered a standardised test battery (as detailed in Duffy et al., 2014), assessing processing speed, learning, memory, language, visuospatial skills, and executive functions. For descriptive purposes, the Wechsler Test of Adult Reading (WTAR) Full Scale IQ (Wechsler, 2001) was administered as an estimate of premorbid functioning. While not the focus of this study, neuropsychological test results were used to inform diagnostic classifications of SCD, MCI, or dementia. These were conducted via consensus of a neurologist or geriatrician and two clinical neuropsychologists, according to established clinical criteria (e.g., Winblad et al., 2004).

#### 2.2.2 Self‐report questionnaires

One week prior to attending the clinic, all participants received a hard copy or digital link to a set of self‐report questionnaires to complete, including the 15 item Geriatric Depression Scale‐15 (GDS‐15; Sheik & Yesavage, 1986), as well as several sleep questionnaires, as follows.

##### **CogSleep** Screener

The CogSleep Screener was developed by sleep and cognition clinical researchers (SLN, LM) and was designed as a brief, 10‐item self‐report questionnaire measuring various aspects of an individual's subjective sleep quality and behaviours for the past 7 days. Items probe common features of sleep disturbances observed in older people and particularly in early or prodromal dementia. In the first three items, participants are asked to record their sleep habits. Items four to seven then elicit the frequency (ranging from 0 to 7 nights/days) that they experienced sleep disturbances, symptoms of RBD, and/or daytime sleepiness over the preceding 7 days. The total score was initially calculated as the sum of each question (maximum total score = 79), with higher scores indicating poorer sleep quality. The CogSleep Screener is shown in Appendix A.

##### Pittsburgh sleep quality index ( **PSQI** )

The PSQI is a 19‐item measure of overall subjective sleep quality. It probes for disturbances over the previous month. Most questions are rated on a 4‐point Likert scale (Buysse et al., 1989) which are summarised into a global score based on the seven components of sleep: quality, latency, duration, efficiency, disturbances, as well as use of sleeping aids and daytime dysfunction. A maximum global score of 21 indicates severe sleep difficulties in all areas, while a score greater than five suggests substantial sleep disturbances in a minimum of two domains, or modest disturbances in more than three domains. The PSQI was developed for use in clinical psychiatric and research populations and is widely used, though it has not yet been specifically validated for use in memory clinic settings.

##### **REM** behaviour disorder screening questionnaire ( **RBDSQ** )

The RBDSQ is a validated, diagnostic, 10‐item self‐rated, “yes/no” questionnaire to screen for the presence of RBD (Stiasny‐Kolster et al., 2007). The questionnaire assesses dream frequency and subject, and their association with nocturnal movements, behaviour, awakenings, and neurological disorders. The maximum score is 13 points and a total of ≥5 indicates idiopathic RBD. The RBDSQ was developed for use in sleep clinic settings and with healthy controls. It has been widely used, and recommended for use in Parkinson's disease clinics (Nomura et al., 2011), but has not been validated for use in memory clinic settings.

##### Insomnia severity index ( **ISI** )

The ISI is a 7‐item scale that evaluates: sleep onset severity, sleep maintenance, early morning awakenings, sleep pattern satisfaction, interference with daily functioning, obviousness of impairment due to the sleep problem, and level of distress caused by sleep disturbance (Morin, 1993). Each item is assessed based on the past 2 weeks and ranked on a 5‐point Likert scale. The maximum score is 28, which indicates the highest level of insomnia severity. The ISI has been validated for use in insomnia populations ranging from 17 to 84 years of age and has been widely used, though does not appear to have been validated for use in memory clinic settings. A score between 0 and 7 indicates no clinically significant insomnia, a score of 8–14 indicates subthreshold insomnia, a score between 15 and 21 indicates clinical insomnia (moderate severity), and a score between 22 and 28 indicates clinical insomnia (severe). A cut‐off score of 10 is recommend in the community sample (Morin et al., 2011).

##### Sleep disturbances group classification

To classify participants into those with or without sleep disturbances, we used the suggested cut‐off scores from the three established sleep questionnaires (i.e., PSQI total ≥5, RBDSQ ≥5, ISI ≥10).

### 2.3 Statistical analyses

All analyses were conducted using the Statistical Package for the Social Sciences (SPSS) for Windows, version 25 (IBM, 2019) and Python (version 3.12.2). In Python, Pandas (version 2.2.2) and NumPy (version 1.26.4) were utilised for Confirmatory Factor Analysis (CFA) and Parallel Analysis (PA). Prior to analyses, all data were visually inspected using frequency tables and boxplots to detect potential outliers or data entry errors. Suspected values and potential outliers were checked against the source document and corrected if necessary. For all tests, the alpha level was set at 0.05 (two‐sided) and non‐parametric tests were used where data violated assumptions of normality.

#### 2.3.1 Sleep habits

The first three items of the CogSleep Screener probed usual bedtime, usual wake up time, and minutes to fall asleep over the past week. These three questions were used to estimate total sleep time with the following formula:

$$\text{Total sleep time}=\begin{array}{l} \text{Usual wake}\;\mathrm{up}\;\text{time}-\text{usual bedtime}\\ -\;\text{minutes to fall asleep. } \end{array}$$

While this is not the conventional way of capturing total sleep time (i.e., using a single item such as the question 4 from PSQI which asks the participants' actual sleep time), we wished to determine if using this formula would achieve similar results, and hence reduce the need to ask an additional question, thereby making the instrument more succinct. Therefore, the estimated total sleep time derived from the CogSleep Screener was first compared with the estimated total sleep time computed using the same formula with PSQI questions 1 to 3. Then, we also examined its correlation with PSQI question 4.

#### 2.3.2 Components of sleep disturbance

For the remaining seven items, principal component analysis with varimax rotation was employed to assess the factor structure of CogSleep Screener items. Items that loaded on the same component were categorised within the same clinical subdomain. Subdomain scores were then calculated as the average of all items within that subdomain, to account for a possible difference in the number of items making up each subdomain.

#### 2.3.3 Psychometric properties

To determine the suitability of the dataset for factor analysis, both the Kaiser‐Meyer‐Olkin (KMO) measure of sampling adequacy and Bartlett's test of sphericity were conducted. To explore the factors of the CogSleep Screener, we randomly divided our sample into calibration and validation samples. Initially, in the calibration sample, we conducted parallel analysis to determine the optimal number of factors to retain. Subsequently, we performed exploratory factor analysis (EFA) using the Principal Axis Factoring method with Varimax rotation, based on the number of factors identified from parallel analysis. Following this, we used the validation sample to conduct confirmatory factor analysis (CFA) using Maximum Likelihood Estimator (MLE) to validate the model identified in the calibration sample.

To test the convergent validity of the CogSleep Screener, Spearman correlations were used to examine the relationship between CogSleep Screener subdomain scores corresponding questionnaires (i.e., ISI, RBSDQ, and PSQI). Internal consistency of the CogSleep Screener was assessed using Revelle's Omega coefficient (McNeish, 2018; Xiao and Hau, 2023). For discriminant validity, we stratified participants into two groups based on the cut‐off for sleep disturbance for ISI (Morin, 1993) and RBDSQ (Stiasny‐Kolster et al., 2007). Since there is no existing cut‐off for daytime sleepiness (i.e., PSQI component 7), we performed a median split to separate participants into those with higher daytime sleepiness and lower daytime sleepiness. In terms of discriminant validity, receiver operating characteristic curve statistics were calculated for the CogSleep Screener and tested against the corresponding sleep screening questionnaire in the whole sample and within specific diagnostic groups (i.e., SCD, MCI, and early dementia). Published rules were followed when interpreting the area under the curve (AUC) (Hosmer Jr et al., 2013). Subsequently, one‐way analysis of covariance (ANCOVA) was used to explore group differences on the CogSleep Screener whilst accounting for differences in key demographic/clinical variables (e.g., age). [Correction added on April 2025, after first publication: The method to assess internal consistency has been changed from using the Cronbach's alpha to Revelle's Omega coefficient. Parallel analysis was added to determine the optimal number of factors to retain.]

## 3 RESULTS

### 3.1 Sample characteristics

In total, 497 clinic participants completed the CogSleep Screener. Table 1 shows sample characteristics, including age, sex, education, MMSE, WTAR, CIRS‐G, GDS‐15, PSQI, RBDSQ, and ISI. On average, participants were 67.72 years old (range = 50–89) with 65.0% (*n* = 323/497) aged over 65 years. They were well educated (mean = 14.32 years). A mean MMSE score of 28.39 suggested normal global cognitive functioning on this gross screening assessment. However, the more detailed neuropsychological and clinical assessment with clinical consensus revealed that 38.2% of the cohort was classified with SCD, 50.5% with MCI, and 11.3% with early dementia (7.4% of which 3.9% were AD and 4.5% were mixed or other dementias). Mean GDS‐15 scores indicated generally low depressive symptoms (3.79), although 24.3% (121/497) scored >5 indicating possible depression. The average PSQI total score was 6.80 out of 21 (59.0% > cut‐off score of 5), and the average ISI total score was 7.50 (29.0% > cut‐off score of 10), both reflecting some degree of sleep disturbance in this sample. Participants were in the ‘normal’ range of scores on RBDSQ (mean = 2.65). In terms of alcohol and smoking, participants reported consuming 5.11 drinks per week on average, and 43.3% reported a smoking history. Ninety nine (19.9)% and 20 (4.0%) of the participants were taking new generation antidepressants and benzodiazepines, respectively.

**TABLE 1:** Demographic and clinical characteristics of the study sample, mean (SD).

	SCD (*n* = 190)	MCI (*n* = 251)	Dementia (*n* = 56)	Total (*N* = 497)
Age, years	65.90 (7.87)	68.20 (8.37)	71.77 (7.20)	67.72 (8.24)
Sex, female [n (%)]	131 (69.0)	156 (62.1)	30 (53.5)	256 (63.8)
Education, years	14.34 (2.62)	14.71 (2.95)	12.37 (3.19)	14.31 (2.94)
Body Mass Index	26.85 (5.05)	27.06 (5.27)	26.98 (4.49)	26.298 (5.10)
MMSE, /30	29.25 (1.08)	28.52 (1.93)	24.86 (2.76)	28.41 (2.19)
WTAR FSIQ	105.47 (7.82)	107.32 (7.71)	102.56 (15.73)	106.10 (9.05)
CIRS‐G, /56	1.30 (0.54)	1.39 (0.44)	1.61 (0.53)	1.38 (0.50)
GDS‐15, /15	4.07 (3.42)	3.57 (3.63)	3.80 (3.47)	3.79 (3.54)
PSQI, /21	7.77 (3.84)	6.59 (3.56)	4.41 (2.68)	6.80 (3.71)
RBDSQ, /13	2.74 (2.23)	2.67 (2.46)	2.24 (2.70)	2.65 (2.40)
ISI, /28	8.54 (5.88)	7.58 (5.86)	3.44 (3.93)	7.50 (5.87)
Alcohol, drinks per week	4.63 (5.77)	4.94 (6.65)	6.49 (7.32)	4.99 (6.41)
Smoking history, n (%)	79 (53.0)	108 (49.1)	24 (45.3)	211 (50.0)
Antidepressants, n (%)	36 (19.1)	57 (23.1)	6 (11.0)	99 (20.2)
Benzodiazepines, n (%)	9 (4.8)	9 (3.6)	2 (3.6)	20 (4.0)

*Note:* Values are mean (standard deviation).

Abbreviations: MMSE, Mini‐Mental State Examination; WTAR FSIQ, Wechsler Test of Adult Reading – Full scale IQ; CIRS‐G, Cumulative Illness Rating Scale – Geriatric version; GDS‐15, Geriatric Depression Scale‐15 (short version); PSQI, Pittsburgh Sleep Quality Index; RBDSQ, REM Behaviour Disorder Screening Questionnaire; ISI, Insomnia Severity Index.

[Correction added on April 2025, after first publication: The table has been updated to reflect the updated demographic and clinical characteristics of the sample study.]

For descriptive purposes, sleep quality data captured from questions 4–10 are shown in Table 2. Across all items except for the questions relating to RSBD symptoms (i.e., Q7 & 8), 50–62% of the participants reported sleep disturbances for at least 1 night of the week. This proportion was similar in those with SCD (54%–70%) and MCI (49%–63%).

**TABLE 2:** The percentage of participants reporting sleep disturbances across the week^a^.

		SCD (*n* = 190)^b^		MCI (*n* = 251)^b^		Dementia (*n* = 56)^b^		Total (*n* = 497)	
		0 days	1–7 days	0 days	1–7 days	0 days	1–7 days	0 days	1–7 days
Q4. Taking more than 30‐minutes to fall asleep at night?	%	46	54	49	51	70	30	50	50
	N	88	102	123	128	39	17	250	247
Q5. Waking during the night and finding it difficult to fall asleep again?	%	31	69	37	63	61	39	37	63
	N	59	131	92	159	34	22	185	312
Q6. Waking up too early in the morning and not being able to fall asleep again?	%	33	67	43	57	64	36	42	58
	N	62	128	109	142	36	20	207	290
Q7. Having vivid dreams, or acting out your dreams (e.g. punching, kicking, screaming)?	%	75	25	81	19	82	18	79	21
	N	143	47	202	49	46	10	391	106
Q8. Experiencing nightmares or frightening dreams?	%	79	21	82	18	86	14	81	19
	N	150	40	206	45	48	8	404	93
Q9. Feeling overly sleepy during the day?	%	30	70	39	61	63	37	38	62
	N	57	133	98	153	35	21	190	307
Q10. Napping during the day?	%	41	59	41	59	46	54	41	59
	N	78	112	102	149	26	30	206	291

^a^ Participant responses to items 4–10 of the CogSleep Screener. Respondents were instructed to answer the next questions relate to their sleep quality over the past week, and mark on the scale the number of nights/days they experienced. The data was then analysed according to either 0 days or 1–7 days.

^b^ Deviations from total *n* or % <100 are due to missing data.

[Correction added on April 2025, after first publication: The table has been updated to reflect the updated percentage of participants reporting sleep disturbances across the week.]

### 3.2 Factor structure of the **CogSleep** Screener

The obtained KMO value of 0.62 and Bartlett's test p‐value (< 0.01) showed the dataset was suitable for factor analysis. The parallel analysis results from the calibration sample indicated that the 7 items of the CogSleep Screener (i.e., question 4 to question 10) supported a 3‐factor solution (Horn, 1965). The EFA result showed three factors explained 73.79% of the variance in total CogSleep Screener score. Table 3 displayed the magnitude of each factor loading. According to Table 3, items were categorised into three different subdomains: *Insomnia* (CogSleep Screener question 4, 5, and 6), *REM Sleep Behaviour Disorder symptoms* (RSBD; CogSleep Screener question 7 and 8), and *Daytime Sleepiness* (CogSleep Screener question 9 "feeling sleepy" and 10 "napping"). The CFA conducted on the validation sample indicates a well‐fitting model with χ^2^(11) = 40.612, *p* < 0.01, the Root Mean Square Error of Approximation (RMSEA) = 0.10, the Standardized Root Mean Square Residual (SRMR) = 0.068, and the Comparative Fit Index = 0.94. In terms of group differences of the subdomain scores, Table 4 shows that the average scores of the *Insomnia*, *RSBD*, and *Daytime Sleepiness* subdomains for the whole sample were 1.56, 0.38, and 1.76, respectively. As the clinical group has accounted for cognition, we only included age as a covariate in the ANCOVA which showed that SCD and MCI groups subjectively reported significantly greater *Insomnia* severity compared with the dementia group. However, there was no significant difference between the clinical groups for the other two subdomains.

**TABLE 4:** Group differences of the CogSleep Screener subdomain scores.

Subdomain scores	SCD (*n* = 190)	MCI (*n* = 251)	Dementia (*n* = 56)	Total (*n* = 497)	*F*	*p*	Group differences
Insomnia (Qs 4, 5, 6, range 0–7)	1.80 (1.66)	1.56 (1.62)	0.72 (1.01)	1.56 (1.61)	10.43	**<0.001**	DEM < SCD, MCI
REM Pathology (Qs 7, 8, range 0–7)	0.45 (0.89)	0.34 (0.79)	0.33 (1.03)	0.38 (0.86)	1.26	0.29	N/A
Daytime sleepiness (Qs 9, 10, range 0–7)	1.93 (1.77)	1.68 (1.67)	1.56 (1.98)	1.76 (1.74)	2.41	0.09	N/A

*Note*: SCD, Subjective cognitive impairment; MCI, Mild Cognitive Impairment; Dem, Dementia; Qs, questions on the specific questionnaire comprising the subdomain. [Correction added on April 2025, after first publication: The table has been updated to reflect the updated group differences of the CogSleep Screener subdomain scores.]

**FIGURE 1:**
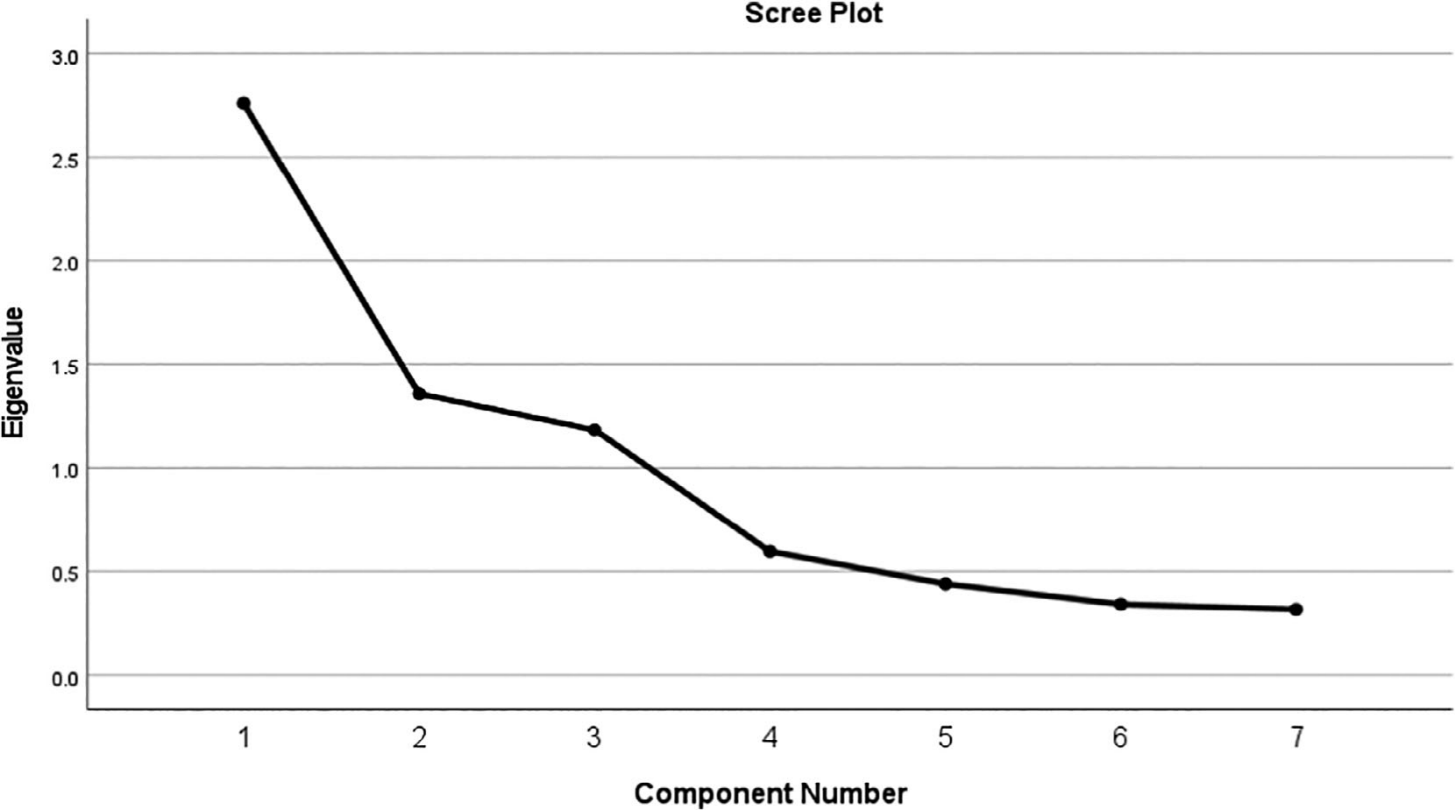
The Scree plot of the exploratory principal component analysis of the 7 items (question 4–10) from the CogSleep Screener.

**TABLE 3:** Factor loading estimates from model in the calibration samples (*n* = 249)

	Factor 1	Factor 2	Factor 3
Q4	0.78		
Q5	0.85		
Q6	0.77		
Q7		0.91	
Q8		0.89	
Q9			0.73
Q10			0.83

*Note:* Factor 1, Insomnia; Factor 2, REM Pathology; Factor 3, Daytime Sleepiness

[Correction added on April 2025, after first publication: This is a new table added to illustrate the factor loading estimates from model in the calibration samples.]

### 3.3 Acceptability of the questionnaire

Ninety nine percent (495/497) of consecutively recruited participants completed the CogSleep Screener in full, indicating high acceptability.

#### 3.3.1 Convergent validity

In terms of convergent validity, the estimated total sleep time of the CogSleep Screener was significantly correlated to the estimated total sleep time from the PSQI (Spearman rho = 0.703, *p* < 0.01), as well as the total sleep time reported in PSQI question 4 (Spearman rho = 0.321, *p* < 0.001). The *Insomnia* subdomain was significantly correlated with ISI (Spearman rho = 0.338, *p* < 0.001). The *RSBD* subdomain was significantly correlated with RBDSQ (Spearman rho = 0.483, *p* < 0.001), and lastly, the *Daytime Sleepiness* subdomain was significantly correlated with PSQI component 7 (Spearman rho = 0.379, *p* < 0.001).

#### 3.3.2 Internal consistency

The Revelle's Omega total for the CogSleep Screener was 0.74, reflecting acceptable internal consistency. For the Insomnia subdomain, which encompasses questions 4 through 6, the Omega was 0.78. The RSBD subdomain, covering questions 7 and 8, showed an Omega of 0.77. The Daytime Sleepiness subdomain, which includes questions 9 and 10, returned a slightly lower Omega of 0.68.

#### 3.3.3 Discriminant validity

In the whole sample, the *Insomnia* subdomain score showed good discriminative ability between participants without insomnia and participants with insomnia using a cut‐off score of 10 (AUC = 0.85, *p* < 0.01), with an optimal cut‐off score of 0.35 showing 76% sensitivity and 80% specificity as shown in Supplementary Table S1A. The *Insomnia* subdomain score showed a similar discriminative ability in SCD with AUC = 0.830 (*p* < 0.01) and MCI with AUC = 0.866 (*p* < 0.01), compared with the dementia subgroup with AUC = 0.613 (*p* = 0.51). Despite the AUC for dementia subgroup suggesting a good discriminant ability, the *p*‐value was not significant possibly due to the small sample size (*n* = 55) and only 2 participants with insomnia were assessed with the ISI.

The *RSBD* subdomain score showed good discriminative ability between participants scoring positive on the RSBDQ vs. those scoring negative (AUC = 0.796, *p* < 0.01), with an optimal cut‐off score of 0.11 showing 76% sensitivity and 78% specificity as shown in Supplementary Table S1B. In respect to the diagnostic subgroups, the *RSBD* subdomain showed consistently good discriminant validity in SCD (AUC = 0.779, *p* < 0.01), MCI (AUC = 0.812, *p* < 0.01), and dementia (AUC = 0.773, *p* = 0.03).

Lastly, the *Daytime Slee*p*iness* subdomain score showed good discriminative ability between participants without daytime sleepiness and those scoring above the median of PSQI component 7 (AUC = 0.73, *p* < 0.01), with an optimal cut‐off score of 0.16 showing 71% sensitivity and 66% specificity as shown in Supplementary Table S1C. The *Daytime Sleepiness* subdomain also showed constantly good discriminant validity in SCD (AUC = 0.788, *p* < 0.01) and MCI (AUC = 0.675, *p* < 0.01), but was not significant in dementia (AUC = 0.590, *p* = 0.47), possibly due to the small sample of people reporting daytime sleepiness. [Correction added on April 2025, after first publication: Due to the updated participant data, sample characteristics, and statistical method, and the associated statistics have been updated.]

## 4 DISCUSSION

The CogSleep Screener is a quick and easy self‐report sleep instrument specifically designed for older adults presenting for assessment of cognitive concerns and at‐risk of, or with early dementia. The instrument covers three domains of sleep disturbances: *Insomnia*, *RSBD*, and *Daytime Slee*p*iness*. Overall, we demonstrated that the instrument possesses appropriate psychometric properties for older adults aged over 50 presenting to a memory and cognition clinic setting.

The 10 items of the questionnaire were conceptualised with reference to currently utilised self‐report instruments including the PSQI, RBDSQ, and ISI, each of which probe distinct aspects of sleep disturbances. Accordingly, EFA revealed a three‐factor solution loaded onto the three subdomains we expected from each questionnaire – *Insomnia* (from ISI and PSQI), *RSBD* (from RBDSQ) and *Daytime Slee*p*iness* (from PSQI component 7). Both the *Insomnia* (r > 0.70) and *RSBD* (r > 0.48) subdomains correlated strongly with the corresponding elements from the original questionnaires, demonstrating good convergent validity. In comparison, the *Daytime Slee*p*iness* subdomain only achieved a moderate correlation (r = 0.40) likely due to the slight differences in capturing daytime dysfunction (PSQI) and daytime sleepiness (CogSleep Screener). That is, the two items used to indicate daytime dysfunction in PSQI probe whether the person can maintain daytime functioning relevant to sleep, whereas questions 9 and 10 in the CogSleep Screener probe for daytime sleepiness (e.g., “need for naps”). The internal consistency is acceptable (ω = .74) and comparable to the validation of other sleep instruments which ranged from α = 0.72 to 0.79 (Hanish et al., 2017; Sancho‐Domingo Winer et al., 2021).

Furthermore, the total sleep time captured by the first three items of both the PSQI and the CogSleep Screener are highly consistent (r = 0.71). Given this is not the traditional way to capture self‐reported total sleep time, we also sought to determine if the estimated total sleep time using only the first three items of the CogSleep Screener (i.e., sleep onset, sleep offset & sleep latency), was correlated with an item from the PSQI (i.e., Q4) that specifically asks about hours of sleep per night. The moderate significant correlation (*r* = 0.32) might be attributed to the two questionnaires examining different sleep periods (i.e., PSQI – for the past month, CogSleep Screener – for the past week). Further work with objective sleep measures would be needed to determine if total sleep time might be best captured by a single item (e.g., Q4 in PSQI) or can be calculated from other measures.

Older adults from the HBA clinic had moderate levels of sleep disturbance, which is consistent with prior work in memory clinic settings (McKinnon et al., 2014). However, one study showed that only 34% of 141 memory clinic clinicians use sleep scales in their practice (Mehrani et al., 2021), possibly due to low time, burden for patients, or unfamiliarity with the variety of sleep presentations associated with neurocognitive syndromes. Currently, to probe for sleep disorders in clinical settings, a number of disparate self‐report measures are required. While brief questionnaires have been developed for other settings (e.g., mental health, Sancho‐Domingo et al., 2021), they do not yet exist for use in memory clinics. We now propose that the CogSleep Screener could be used in this setting. It has the advantage of assessing all three sleep domains in one brief instrument within the same timeframe (the prior week). It can be used prior to clinical examination without additional/in‐depth training and the high patient completion rates are promising for implementation purposes. The outcome of the questionnaire would assist in identifying specific types of sleep disturbances referral for more thorough examination of sleep such as via PSG. Indeed, the actual diagnoses of sleep orders such as RBD require video confirmation with PSG (Cesari et al., 2021). However, the CogSleep Screener assesses sleep for a week, whereas PSG‐based RBD diagnosis often rely on sleep monitoring of one night which has been shown to be problematic (Wohlgemuth et al., 1999). As such, this instrument could serve as a time‐effective tool to assess the need for further sleep assessments which would enhance the cost‐effectiveness of sleep screening in the memory and cognition clinic setting.

### 4.1 Further developments

Whilst the current version of the CogSleep Screener addresses more elements of sleep disturbances than most existing self‐report sleep questionnaires for older adults, it does not probe for other sleep disorder pathologies such as obstructive sleep apnea, restless leg syndrome (the urge to move legs to relieve unpleasant sensations), or narcolepsy (chronic neurological disorder that affects the brain's ability to control sleep–wake cycles). This is because current self‐report based approaches for detection of these elements lack sophistication for use in cognitively impaired individuals (Fulda et al., 2021; Wilson et al., 2014), and may require the participant to enter values such as neck circumference, blood pressure, and body mass index (Martins et al., 2020), and/or the use of clinical interview. Thus, questionnaire development for such disorders requires more efforts. In the interim, objective measures such as PSG (Masa et al., 2013; Michaud et al., 2002) or the use of appropriately validated wearable devices are still advised.

Sleep quality is another key convergent that has the potential to be added to the future version of the questionnaire with just one item. Sleep quality is a core component of insomnia and studies support a relationship between sleep quality and various biomarkers and functional outcomes for neurodegeneration including cognition, beta‐amyloid, chronic health conditions, and mortality (Lucey et al., 2021; Zhong et al., 2015). Indeed, sleep quality and daytime dysfunction are key factors that might determine health seeking behaviour. In addition, while the current questionnaire includes sleep onset and offset, which are closely aligned with gold‐standard circadian measures (e.g., melatonin (Lewy et al., 1999)), assessment of chronotype (e.g., via the Horne & Ostberg Morningness‐Eveningness Questionnaire (Horne & Ostberg, 1976)) could be worth considering as an additional measure when assessing circadian misalignment (phase advance or delay).

### 4.2 Limitations and conclusions

Within the scope of this study, test–retest reliability was not examined. Furthermore, while the scale benefits from brevity, it is usually more ideal to have more than two items for each factor. While a strength of the CogSleep Screener is the use of one common recent timeframe (i.e., the past week), the validation questionnaires used varied timeframes (PSQI = the past month, ISI and RBDSQ = the past 2 weeks). Due to limited sample size (for the dementia subgroups), the results for ROC analyses should be interpreted with caution. The variability in reporting of sleep disturbance over periods of weeks to months is unknown in cognitively impaired individuals, although difficulties with memory are factors to consider in all measures requiring the participant to recall prior experiences and events (Blackman et al., 2023). It is noted that while this study included some individuals with mild dementia, it may not be suitable for those with an MMSE <20, and therefore we suggest further validation of this tool for those with more advanced cognitive impairment. Lastly, future studies should also be required for examine whether the scale measures the constructs comparably across those from varied ethnic or culturally and linguistically diverse backgrounds., as well as in those meeting clinical criteria for sleep disorders, through factorial invariance with larger sample sizes and different cohort worldwide. [Correction added on April 2025, after first publication: The associated statistics have been updated to reflect the updated participants data]

## 5 CONCLUSION

In conclusion, the CogSleep Screener is suitable for use in the memory and cognition clinic setting for those with MMSE of ≥20. It has the potential to be used as a frontline screening instrument to triage sleep disturbances for all types of healthcare providers in primary, secondary, and tertiary care settings as well as within residential aged care facilities. The addition of objective measures may still be required for some disorders such as sleep apnea, restless legs, and narcolepsy. Future revisions should incorporate daytime dysfunction and sleep quality. Further work could also examine whether the subdomains that predict sleep health and/or cognitive outcomes, are reliable over time and whether the instrument and/or the subdomains are sufficiently sensitive to the effect of interventions. Finally, feasibility work seeking to implement the use of sleep screening within a broader array of memory clinic settings is now required.

## AUTHOR CONTRIBUTIONS

**Simone Simonetti:** Writing – original draft; conceptualization; formal analysis. **Shawn Kong:** Conceptualization; writing – original draft; methodology. **Zoe Menczel Schrire:** Writing – original draft; conceptualization; data curation. **Ping Hsiu Lin:** Writing – original draft; formal analysis; visualization. **Nathan Cross:** Writing – review and editing. **Loren Mowszowski:** Writing – review and editing; conceptualization; supervision. **Catriona Ireland:** Writing – review and editing. **Ivana Rosenzweig:** Writing – review and editing. **Sharon L. Naismith:** Supervision; funding acquisition; writing – review and editing; conceptualization; methodology; writing – original draft.

## FUNDING INFORMATION

This work was supported by the National Health and Medical Research Council (NHMRC) Centre of Research Excellence to Optimise Sleep in Brain Ageing and Neurodegeneration (CogSleepCRE GNT1152945); NHMRC – Australian Research Council Dementia Research Development Fellowship [1109618]; NHMRC – Boosting Dementia Research Leadership 2 Fellowship [2008064].

## PATIENT CONSENT STATEMENT

Written informed consent was obtained from all subjects involved in the study.

[Correction added on April 2025, after first publication: (Horn 1965), (McNeish 2018), and (Xiao and Hau 2023) have been added to the reference list.]

## Supporting information


**TABLE S1A.** Sensitivity and specificity of the CogSleep Screener *Insomnia* subdomain scores (Qs 4, 5, and 6) against ISI subthreshold insomnia.

**TABLE S1B.** Sensitivity and specificity of the CogSleep Screener *Rapid Eye Movement Symptoms* subdomain scores (Qs 7 and 8) against RSBDQ.

**TABLE S1C.** Sensitivity and specificity of the CogSleep Screener *Daytime Sleepiness* subdomain scores (Qs 9 and 10) against PSQI component 7.

## Data Availability

The data that support the findings of this study are not available to be shared due to privacy or ethical restrictions from the affiliated institution.

## APPENDIX A

### A.1 The CogSleep Screener – Patient Version

**Table:**

The following questions relate to your usual sleep habits over the last week.							
1.	What time do you typically go to bed at night?				Time…………. pm		
2.	What time do you typically wake up in the morning?				Time…………. am		
3.	On average, how long does it take for you to fall asleep?				……….… mins		
The next questions relate to your sleep quality over the last week. Please circle the number of nights/days you experienced the following:							
4. Taking more than 30‐minutes to fall asleep at night?							
0	1	2	3	4	5	6	7
5. Waking during the night and finding it difficult to fall asleep again?							
0	1	2	3	4	5	6	7
6. Waking up too early in the morning and not being able to fall asleep again?							
0	1	2	3	4	5	6	7
7. Having vivid dreams, or acting out your dreams (e.g., punching, kicking, screaming)?							
0	1	2	3	4	5	6	7
8. Experiencing nightmares or frightening dreams?							
0	1	2	3	4	5	6	7
9. Feeling overly sleepy during the day?							
0	1	2	3	4	5	6	7
10. Napping during the day?							
0	1	2	3	4	5	6	7

### A.2. Scoring (office use only):

**Table:**

Domain	Calculation	Cut‐score*	Above threshold?
Insomnia	Qs (4 + 5 + 6)/3	0.35	Y/N
Rapid eye movement symptoms	Qs (7 + 8)/2	0.11	Y/N
Daytime sleepiness	Qs (9 + 10)/2	0.16	Y/N

*Note*: *Scores above the cut‐score indicate the presence of insomnia/rapid eye movement symptoms/daytime sleepiness.

[Correction added on April 2025, after first publication: The appendix has been updated to reflect the new cut‐score of the instrument.]
