# Supplementary material for: Validating the CogSleep Screener in older adults at a memory and cognition clinic
Source: J Sleep Res. 2024 Sep 30;34(3):e14355. doi: 10.1111/jsr.14355 (PMC12069752; doi:10.1111/jsr.14355)
Supplement: Supplementary file 1 — TABLE S1A. Sensitivity and specificity of the CogSleep Screener Insomnia subdomain scores (Qs 4, 5, and 6) against ISI subthreshold insomnia. [file JSR-34-e14355-s003.docx]

| **Supplementary Table 1a. Sensitivity and specificity of the CogSleep Screener *Insomnia* subdomain scores (Qs 4, 5, and 6) against ISI subthreshold insomnia.** | | |
| --- | --- | --- |
| Scores | Sensitivity | Specificity |
| 0.15 | 0.93 | 0.44 |
| **0.35** | **0.76** | **0.80** |
| 0.43 | 0.69 | 0.86 |
| 0.52 | 0.62 | 0.91 |
| 0.69 | 0.47 | 0.94 |
| 0.86 | 0.28 | 0.98 |
| 1.00 | 0.01 | 0.99 |

[Correction added on April 2025, after first publication: The table has been updated to reflect the updated participants data.]
