# Supplementary material for: Validating the CogSleep Screener in older adults at a memory and cognition clinic
Source: J Sleep Res. 2024 Sep 30;34(3):e14355. doi: 10.1111/jsr.14355 (PMC12069752; doi:10.1111/jsr.14355)
Supplement: Supplementary file 2 — TABLE S1B. Sensitivity and specificity of the CogSleep Screener Rapid Eye Movement Symptoms subdomain scores (Qs 7 and 8) against RSBDQ. [file JSR-34-e14355-s002.docx]

| **Supplementary 1b. Sensitivity and specificity of the CogSleep Screener *Rapid Eye Movement Symptoms* subdomain scores (Qs 7 and 8) against RSBDQ.** | | |
| --- | --- | --- |
| Scores | Sensitivity | Specificity |
| 0.07 | 1.00 | 0.0 |
| **0.11** | **0.76** | **0.78** |
| 0.16 | 0.60 | 0.89 |
| 0.32 | 0.22 | 0.96 |
| 0.66 | 0.09 | 0.99 |
| 1 | 0.01 | 0.99 |
|  |  |  |

[Correction added on April 2025, after first publication: The table has been updated to reflect the updated participants data.]
