# Supplementary material for: Validating the CogSleep Screener in older adults at a memory and cognition clinic
Source: J Sleep Res. 2024 Sep 30;34(3):e14355. doi: 10.1111/jsr.14355 (PMC12069752; doi:10.1111/jsr.14355)
Supplement: Supplementary file 3 — TABLE S1C. Sensitivity and specificity of the CogSleep Screener Daytime Sleepiness subdomain scores (Qs 9 and 10) against PSQI component 7. [file JSR-34-e14355-s001.docx]

| **Supplementary Table 1c. Sensitivity and specificity of the CogSleep Screener *Daytime Sleepiness* subdomain scores (Qs 9 and 10) against PSQI component 7.** | | |
| --- | --- | --- |
| Scores | Sensitivity | Specificity |
| 0.10 | 0.84 | 0.42 |
| **0.16** | **0.71** | **0.66** |
| 0.23 | 0.52 | 0.79 |
| 0.49 | 0.13 | 0.97 |
| 0.66 | 0.06 | 0.99 |
| 1.00 | 0.06 | 0.99 |
|  |  |  |

[Correction added on April 2025, after first publication: The table has been updated to reflect the updated participants data.]
